# Randomized controlled trials of mind–body interventions for posttraumatic stress disorder: a systematic review

**DOI:** 10.3389/fpsyg.2023.1219296

**Published:** 2024-01-24

**Authors:** Josh Kaplan, Vanessa C. Somohano, Belle Zaccari, Maya E. O’Neil

**Affiliations:** ^1^Department of Neurology, Oregon Health & Science University, Portland, OR, United States; ^2^Veterans Affairs Portland Health Care System, Portland, OR, United States; ^3^Department of Psychiatry, Oregon Health & Science University, Portland, OR, United States; ^4^Department of Medical Informatics and Clinical Epidemiology, Oregon Health & Science University, Portland, OR, United States

**Keywords:** mind–body, mindfulness, movement, exercise, intervention, posttraumatic stress disorder, complementary integrative health

## Abstract

Mind–body interventions (MBIs) include mindfulness-based interventions (MiBIs), meditation- and mantra-based interventions (MMIs), and movement-based interventions (MoBIs). These approaches have demonstrated preliminary efficacy in improving posttraumatic stress disorder (PTSD) symptoms. However, previous systematic reviews and meta-analyses have noted that this area of research is limited by inadequate comparator conditions, heterogeneity of measurement, and absence of objective outcome measures. For these reasons, an updated review of the highest-quality evidence available is warranted. We used the Agency for Healthcare Research and Quality (AHRQ)-funded evidence tables for the PTSD-Repository to identify relevant studies and assess the risk of bias as follows: The search was conducted between June 2018 and June 2022, and databases included PTSDpubs (formerly PILOTS), Ovid^®^ MEDLINE^®^, Cochrane CENTRAL, Embase^®^, the Cumulative Index to Nursing and Allied Health Literature (CINAHL^®^), SCOPUS, and PsycINFO^®^. Twenty-six randomized controlled trials met our inclusion criteria. After identifying studies and retrieving risk of bias information from the PTSD-Repository evidence tables, we extracted additional data and synthesized the evidence. The strength of evidence was rated as low for MiBIs and MMIs, largely due to contradicting results, inconsistent use of active versus passive comparators, and high risk of bias. The strength of evidence for MoBIs was rated as moderate due to individual studies consistently favoring the intervention and a relatively large number of studies and participants. Of the 26 included studies, only two included objective outcome measures. Implications for future MBI research and clinical applications for treating PTSD are discussed.

## 1 Introduction

Posttraumatic stress disorder (PTSD) is a health concern with deleterious outcomes (Banks et al., 2015; Hilton et al., 2017; Boyd et al., 2018), with a prevalence of 6% in the United States (Goldstein et al., 2016). The American Psychiatric Association Diagnostic and Statistical Manual of Mental Health Disorders (American Psychiatric Association, 2013) characterizes PTSD by four symptom clusters: intrusions (i.e., intrusive and uncontrollable thoughts, nightmares, and flashbacks), avoidance of internal and external trauma-related stimuli, negative changes in cognitions and mood, and changes in arousal and reactivity.

The Veterans Administration/Department of Defense (VA/DoD) Clinical Practice Guideline recommends evidence-based psychotherapies (EBPs) such as Cognitive Processing Therapy (CPT) and Prolonged Exposure (PE) therapy (VA/DoD Clinical Practice Guideline, 2023). Studies have found support for these interventions in reducing symptoms in veterans (Schnurr et al., 2022). Despite this support, some patients opt not to participate (Hundt et al., 2018), dropout before completing (Lewis et al., 2020), or have residual symptoms after completing CPT or PE (Larsen et al., 2019). For these reasons, recent reviews have suggested the need for alternative treatments (Banks et al., 2015; Boyd et al., 2018). Mind–body interventions (MBIs) may present a viable alternative.

MBIs emphasize the interconnection of the brain, body, behavior, and wellness (Wahbeh et al., 2008) and have gained popularity in treating PTSD. A 2013 review found that 39% of individuals with PTSD utilized alternative treatments, including various MBIs (Libby et al., 2013). For this review, MBIs are described in three categories: mindfulness-based interventions (MiBIs), mantra- and meditation-based interventions (MMIs), and movement-based interventions (MoBIs).

Mindfulness-based interventions (MiBIs) emphasize developing mindfulness, defined as “awareness that emerges through paying attention on purpose, in the present moment” (Kabat-Zinn, 2003). MiBIs may be particularly effective for trauma-related avoidance and negative cognitions (Follette et al., 2006; Lang et al., 2012; Banks et al., 2015; Boyd et al., 2018) and may offer unique benefits by helping regulate physiological systems (Bremner et al., 2017), including those linked to treatment dropout. Indeed, MiBIs show lower average attrition than “first-line” or “gold standard” (VA/DoD Clinical Practice Guideline, 2023) PTSD treatments (Schottenbauer et al., 2008; Goetter et al., 2015; Kehle-Forbes et al., 2016).

Another category of MBIs is meditation- and mantra-based interventions (MMIs). These interventions are typically less structured than MiBIs and emphasize pure meditative practices (Gallegos et al., 2017). Only one known trial has compared an MMI to a “first-line” intervention and found relatively similar outcomes for both interventions (Kearney et al., 2021). More such studies are required to conclude the efficacy of this group of MBIs.

MMIs may result in decreased experiential avoidance and increased ability to approach distress (Vujanovic et al., 2009). Mantra-based meditation increases attention through focus on reciting a word or phrase (Gallegos et al., 2017) and increases mindful attention while reducing PTSD symptoms (Bormann et al., 2014).

Movement-based interventions (MoBIs) include yoga, tai chi, and qi gong. While some studies show promise for MoBIs in treating PTSD (Grodin et al., 2008; Mitchell et al., 2014; Davis et al., 2020), the findings are mixed. Review-level research has found generally insufficient evidence for recommending these treatments for PTSD (Cramer et al., 2018; Niles et al., 2022). MoBIs feature physical postures and breathing practices (Gallegos et al., 2017) and impact PTSD through reduced arousal and increased awareness (van der Kolk et al., 2014), a skill related to emotion regulation and distress tolerance (Gallegos et al., 2017).

“First-line” treatments are effective for many individuals with PTSD. However, given their limitations [e.g., excessive attrition and residual symptoms (Najavits, 2015; Larsen et al., 2019)], a comprehensive understanding of viable alternatives is needed.

Most prior reviews have found preliminary support for MBIs for PTSD, though most qualify these results due to limitations of the overall body of literature (e.g., Banks et al., 2015).

In pursuit of more conclusive results, some recent reviews have limited their scope to methodologically rigorous studies (Polusny et al., 2015; Gallegos et al., 2017; Hilton et al., 2017). However, as a consequence of increased selectivity, the authors of a recent meta-analysis (Gallegos et al., 2017) reported limited sample size, preventing reliable analyses. Additionally, recent reviews have not considered the nature of control conditions, preventing the comparison of MBIs to active treatments for PTSD (Gallegos et al., 2017).

Heterogeneity of measurement is another commonly cited limitation (e.g., Banks et al., 2015; Polusny et al., 2015; Bremner et al., 2017; Hilton et al., 2017). With one exception (Kim et al., 2013), previous reviews have not included physiological outcomes and have not distinguished findings based on self- versus clinician-report measures of PTSD symptoms. For a description of reviews, see Table 1.

**Table 1:** Previous systematic reviews and meta-analyses of MBIs for PTSD.

Citation	Search dates	Number of studies	MBI type	PTSD diagnostic criterion	Study designs	Control conditions	Population
Banks et al. (2015)	1980–2014	12	MiBIs; excluded particular practices.	None	RCT, uncontrolled pilot	WL, TAU, active	Adult sample aged >/= 18
Boyd et al. (2018)	NR (newest 2017)	17	NR; mostly MiBIs with one transcendental meditation.	None	RCT, uncontrolled pilot	TAU, active, NR	Adult sample aged >/= 18
Gallegos et al. (2017)	1946 through 5/31/2016	19	Mind–body, meditation, tai chi, qi gong, yoga, mindfulness, mindfulness-based stress reduction, mindfulness-based cognitive therapy, mantra.	PTSD diagnosis using clinician or self-report measure	RCT	WL, placebo, active	Adults >/= 18 with PTSD
Hilton et al. (2017)	Inception through November 2015	10	Meditation intervention (e.g., MBSR, MBCT, mindfulness meditation, yoga, tai chi, mantra meditation, qigong, and self-compassion).	PTSD diagnosis according to DSM or ICD criteria, or must screen positive for PTSD using a validated measure with symptoms compatible with a PTSD diagnosis	RCT	WL, TAU, active	Adults >/= 18 with PTSD
Hopwood et al. (2017)	NR (newest 2016)	18	MiBIs	No diagnostic criteria Required assessment of PTSD symptoms	RCT	WL, placebo, active	Adult sample aged >/= 18
Kim et al. (2013)	Inception through 6/27/2012	16	MiBIs; physical activities that focus on interaction among brain, body, and behavior, including yoga, tai chi, qigong, MBSR, meditation, and deep breathing.	Included studies of “patients with PTSD” Specific diagnostic criteria NR	RCT, NRS, case study	WL, TAU	Adult sample aged >/= 18
Niles et al. (2018)	1/1985–1/2017	22	Mindfulness, relaxation, yoga, and tai chi interventions	Did not require PTSD diagnosis	RCT	WL, TAU, active	Adult sample aged >/= 18
Wahbeh et al. (2014)	1950–3/12/2013	33	CAM interventions, including MBIs and Natural Products (e.g., herbal and botanical medicines)	Did not require PTSD diagnosis	RCT, uncontrolled	WL, TAU, active, sham, placebo	Adults with PTSD or who were administered PTSD assessment
Taylor et al. (2020)	Inception – 9/2018	66 review 24 meta-analysis	Mindfulness, yoga, tai chi, and qi gong	Did not require PTSD diagnosis assessed MBIs for “consequences of trauma”	RCT, uncontrolled	None, WL, TAU, active	Adults >/= 18 with PTSD

MBI, mind–body intervention; PTSD, posttraumatic stress disorder; MiBI, mindfulness-based intervention; RCT, randomized-controlled trial; WL, waitlist; TAU, treatment-as-usual; NR, not reported; MBSR, mindfulness-based stress reduction; MBCT, mindfulness-based cognitive therapy; DSM, Diagnostic and Statistical Manual; ICD, International Classification of Diseases; NRS, nonrandomized prospective study; CAM, Complementary and Alternative Medicine; CAPS, Clinician-Administered PTSD Scale; MINI, Mini International Neuropsychiatric Interview; WHO, World Health Organization; SCID, Structured Clinical Interview for DSM-IV.

This systematic review addresses the limitations of previous reviews by (1) updating the search to include recent data; (2) focusing on the highest quality evidence; (3) including a comprehensive definition of MBIs, including MiBIs, MMIs, and MoBIs; and (4) evaluating individual studies by control condition (i.e., active versus passive) and utilization of objective outcome measures.

## 2 Method

This systematic review relies on some methods from a recent report from the Agency for Healthcare Research and Quality (AHRQ) on the evidence base for the PTSD-Repository (O'Neil M. E. et al., 2020; O'Neil M. et al., 2020) and follows the AHRQ Methods Guide for Effectiveness and Comparative Effectiveness Reviews (AHRQ Methods for Effective Health Care, 2008). For a description of methods, see O'Neil M. E. et al. (2020); O'Neil M. et al. (2020), and O'Neil et al. (2022). The literature search was scoped for all published, peer-reviewed randomized controlled trials (RCTs) of PTSD and PTSD/substance use disorder treatments in adults (for a comprehensive review of the search strategy, see Appendix A of O’Neil et al., 2023). Electronic databases were searched from 1 June 2018 to 14 June 2022 and included PTSDpubs, Ovid MEDLINE®, Cochrane CENTRAL, Embase®, the Cumulative Index to Nursing and Allied Health Literature (CINAHL®), SCOPUS, and PsycINFO®. PICOTS were used to determine eligibility (see Table 2).

**Table 2:** PICOTS: inclusion and exclusion criteria.

PICOTS	Include	Exclude
Populations	Adults (≥18 years old) with PTSD diagnosed by a clinician or through the administration of a validated clinician-administered or patient-reported assessment tool	Children (<18 years old) Diagnosis of acute stress disorder Studies that do not specify the criteria used to diagnose PTSD
Interventions	Mind–body interventions for PTSD, including mindfulness-based interventions, mediation- and mantra-based interventions, and movement-based interventions	Interventions designed to simultaneously target PTSD and comorbid conditions Pharmacologic interventions Interventions designed to prevent PTSD
Comparators	No limitations applied. Direct head-to-head comparisons of PTSD interventions were included. Interventions such as waitlist, usual care, placebo, or other minimally-active treatment (e.g., education or attention control) are categorized as “Controls”	None
Outcomes	Any overall PTSD outcome	Studies reporting only individual symptoms or symptom clusters without overall PTSD outcome
Timing	Any study duration and length of follow-up	None
Setting	All study settings	None
Study Design	RCTs	Non-RCTs

PICOTS, populations, interventions, comparators, outcomes, timing, setting, study design; PTSD, posttraumatic stress disorder; RCT, randomized-controlled trial.

After screening and inclusion, data were abstracted and compiled into detailed evidence tables (O'Neil et al., 2022). Risk of bias (ROB) evaluations included rating 12 elements according to Cochrane’s Risk of Bias 2.0 methods (Robinson et al., 2014; Forman-Hoffman et al., 2018). An overall rating (low, some concerns, or high) was assigned based on standard ROB domains by one investigator, and another investigator reviewed all ratings; consensus ratings were conducted for any disagreements.

The GRADE (Grading of Recommendations, Assessment, Development, and Evaluations) framework provided criteria for summarizations of evidence (Guyatt et al., 2008a,b, 2011) and was used to evaluate the quality of evidence. GRADE ratings were developed for each intervention category (i.e., MiBIs, MMIs, and MoBIs). Detailed GRADE ratings may be found in Table 3.

**Table 3:** Strength of evidence ratings for intervention categories and comparators.

Intervention group	Comparator type, # Studies (N)	ROB	Findings	SOE (GRADE)
MMIs	Passive, 3 (219)	Low-Some concerns	Favors intervention	Moderate
	Active, 5 (699)			
MiBIs	Passive, 2 (102)	Some concerns-High	Favors intervention	Low
	Active, 6 (501)			
MoBIs	Passive, 7 (631)	Some concerns-High	Favors intervention	Low
	Active, 3 (250)			

MMI, meditation- and mantra-based interventions; MiBI, mindfulness-based interventions; MoBI, movement-based interventions; SOE, strength of evidence; GRADE, Grading of Recommendations, Assessment, Development, and Evaluations.

## 3 Results

Twenty-six studies met the inclusion criteria. All studies reported self- or clinician-reported PTSD outcomes, while three reported physiological outcomes. Table 4 provides study characteristics and findings, which are reviewed in the text below.

**Table 4:** Individual studies and abstracted data from RCTs meeting inclusion criteria.

Citation	Population (*N*)	% Female	Age *Mean*	Age *SD*	Diagnostic method	Trauma type	MBI (*N*)	MBI type	Control (*N*); control type	Main findings
Bellehsen et al. (2022)	Veterans (40)	15	51.6	11.45	CAPS-5	Combat, sexual, disaster, life-threatening/serious injury	TM (20)	MMI	TAU (20); Passive	Significantly greater improvement in clinician-rated (CAPS-5) PTSD symptoms (*p* = 0.012; *d* = −0.84) and in self-reported (PCL-5) PTSD symptoms (*p* = 0.006; *d* = −0.94) in the TM group compared to the control.
Bormann et al. (2013)	Veterans (146)	2.74	57	10.10	CAPS-IV	Combat	MRP + case management (69)	MMI	TAU (73); Passive	Significant improvement in self-report (PCL; *p* < 0.05) and clinician-rated (CAPS; *p* = 0.047) PTSD symptoms in MRP + TAU versus TAU alone.
Bormann et al. (2008)	Veterans (29)	0	56	6.57	CAPS-IV	Combat	Mantra intervention (14)	MMI	Usual care delayed-treatment (15); Passive	Large effect size for improvement in self-report (PCL; Cohen’s *d* = −0.72) PTSD symptoms and small effect size for improvement in clinician reported (CAPS; Cohen’s *d* = −0.33) PTSD symptoms in MMI condition versus control.
Bormann et al. (2018)	Veterans (173)	15	48.9	14.57	CAPS-5	Trauma related to military service	Individual mantram repetition (89)	MMI	Present-centered Therapy (84); Active	Significantly greater improvements in clinician-rated (CAPS-5) score in MMI condition versus control at post-treatment (*p* = 0.006; *d* = 0.49) and at 2-month follow-up (*p* = 0.04; *d* = 0.46). Significantly greater improvement in self-rated (PCL-M) scores in MMI condition versus control at post-treatment (*p* = 0.04; *d =* 0.43), but not at 2-month follow-up (*p* = 0.25, *d* = 0.25).
Bremner et al. (2017)	Veterans (26)	0	34.5	8.5	SCID; CAPS	Combat	MBSR (17)	MiBI	PCGT (9); Active	Significant improvement in clinician-rated (CAPS) avoidance (*p* = 0.026), hyperarousal (*p* = 0.006), and total clinician-rated PTSD symptoms (*p* = 0.016) in MBSR versus control. No significant difference in clinician-rated intrusive symptoms (*p* = 0.14) between MBSR and control.
J.Carter et al. (2013)	Veterans (31)	0	58.5	4.3	CAPS-IV	NR	SKY (16)	MoBI	WL (15); Passive	Significant improvement in self-report (PCLM-17; *p* < 0.01) and clinician-rated (CAPS; *p* = 0.04) PTSD symptoms in yoga condition versus control.
Davis et al. (2019)	Veterans (214)	16.2	51.4	11.2	CAPS-IV	Combat, noncombat, and/or sexual assault	MBSR (96)	MiBI	PCGT (95); Active	Significant improvements in self-report (PCL; *p* = 0.04) PTSD symptoms in MBSR versus PCGT. This difference was non-significant at week 16 follow-up (*p* = 0.68). No significant difference in clinician-administered PTSD symptoms between groups. Significant improvements in clinician-rated (CAPS) PTSD symptoms in both MBSR and PCGT groups.

Citation	Population (*N*)	% Female	Age *Mean*	Age *SD*	Diagnostic method	Trauma type	MBI (*N*)	MBI type	Control (*N*)	Main findings
Davis et al. (2020)	Veterans/community (212)	34.0	50.6	12.9	CAPS-5	Combat, noncombat, and/or sexual assault	HYP (108)	MoBI	WLP (101); Passive	Significant improvements in self-report (PCL-5; *p* = 0.001) and clinician-rated (CAPS-5; *p* < 0.001) PTSD symptoms in HYP versus WLP. Differences were non-significant at 7-month follow-up (*p* = 0.603; *p* = 0.682).
Gallegos et al. (2020)	IPV survivors (29)	100	42.69	13.11	PCL-5	Intimate partner violence	MBSR (19)	MiBI	Active wellness control (10); Active	No significant between-group differences. Significant reductions in symptom severity (PCL-5) in the MBSR condition (*p* < 0.05, *f* = 0.56).
Gibert et al. (2022)	Community (34)	64.7	35	6.25	PCL-5	Terrorist attacks	Meditative scuba diving (Bathysmed; 17)	MoBI	Multisport program (17); active	Non-significant improvement in overall PTSD symptoms in the Bathysmed condition compared to the control. Significant improvement in intrusive symptoms in the Bathysmed condition versus control (*p* < 0.01)
Goldstein et al. (2018)	Veterans (47)	19.15	46.80	14.93	SCID for DSM-IV-TR CAPS for DSM-IV-TR	NR	Integrative exercise (21)	MoBI	WL (26); Passive	Significant reduction in clinician-rated (CAPS) PTSD symptom severity (*d =* −90) in integrative exercise condition relative to control.
Jindani et al., 2015	Community (80)	88.75	Median 41 (range 18 to 64)	–	PCL-17	Mixed	KnY (59)	MoBI	WL (21); Passive	Significantly lower self-reported (PCL-17) PTSD symptoms in the KnY group compared to the waitlist control group at post-treatment (*p <* 0.05).
Kearney et al. (2021)	Veterans (184)	16.3	57.15	13.1	CAPS-5	Combat, sexual assault, other assault, accident, sudden death, other	Loving-kindness meditation (91)	MMI	Cognitive Processing Therapy – Cognitive only (93); Active	In superiority analyses, there were no significant differences between groups on CAPS-5 scores.
Kearney et al. (2013)	Veterans (47)	21.28	52	12.6	PCL-C	NR	MBSR (25)	MiBI	TAU (22); Passive	No significant between-group effects. Significant (*p* = 0.025) and medium-to-large effect size for self-report (PCL) PTSD symptoms in the MBSR condition (d = −0.63).

Study	Population (*N*)	% Female	Age *Mean*	Age *SD*	Diagnostic method	Trauma type	MBI (*N*)	MBI type	Control (*N*)	Main findings
Kearney et al. (2016)	Veterans (55)	14.55	49.9	7.1	PSS-I	NR	MBSR + TAU (26)	MiBI	TAU (29); Passive	Significant improvement in clinician-rated (PSS-I) PTSD symptoms in MBSR +TAU condition versus TAU condition at post-treatment (*p* = 0.001) and follow-up (*p* = 0.02) in completer analysis. Significant improvement in clinician-rated PTSD symptoms in MBSR +TAU condition versus TAU condition at post-treatment (*p* = 0.004), but not at follow-up (*p* = 0.08) in ITT analysis.
Kelly et al. (2021)	Veterans (152)	100	48.38	11.1	MINI 7.0.0	Military sexual trauma	Trauma Center Trauma-Sensitive Yoga (58)	MoBI	Cognitive Processing Therapy (46); Active	Significant group difference for PCL-5 Cluster B [*F* (3,189.4) = 2.84, *p* = 0.039], in which the TCTSY group was superior at mid-course. Clinician-rated (CAPS-5) and self-reported (PCL-5) PTSD symptoms decreased significantly in both groups. This difference was non-significant at 3-month follow-up.
Lang et al. (2019)	Veterans (37)	25.0	49.1	14.5	MINI 6.0; CAPS-5	Combat, violence	Compassion meditation (17)	MMI	VC (20); Active	Significant (*p* = 0.046) improvement in clinician-rated (CAPS-5) PTSD symptoms and large effect size (*d* = −0.85) in the CM group versus VC.
Nidich et al. (2018)	Veterans (202)	16.83	47.0	15.4	CAPS; PCL-M	Mixed	TM (68)	MMI	HE (67); PE (68); Active	Superiority comparisons found significant reductions in clinician-rated (CAPS) PTSD symptoms from baseline to 3-month follow-up for TM compared to PE (*p* = 0.0002) and HE (*p* = 0.0009).
Niles et al. (2012)	Veterans (33)	0	52.0	13.0	CAPS-IV; PCL-M	Combat-related	Telehealth mindfulness (17)	MiBI	Telehealth psychoed. (16); Active	No significant between-group differences. Significant reduction in clinician-rated PTSD symptoms (CAPS) in mindfulness condition (*p* = 0.005). Clinically significant improvement in self-reported PTSD symptoms (PCL-M) in 53.8% of participants in the mindfulness condition.
Polusny et al. (2015)	Veterans (116)	15.52	58.5	9.8	SCID; CAPS-IV; PCL	Mixed	MBSR (58)	MiBI	PCGT (58); Active	Significant reduction in PTSD symptoms (PCL) in MBSR condition versus PCGT at post-treatment (*p* = 0.002) and 2-month follow-up (*p* = <0.001). MBSR participants were more likely to show clinically significant improvement in self-reported PTSD symptoms than PCGT (48.9% vs. 28.1%). No difference between groups in loss of PTSD diagnosis at 2-month follow-up.

Citation	Population (*N*)	% Female	Age *M* (*SD*)		Diagnostic method	Trauma type	MBI (*N*)	MBI type	Control (*N*)	Main findings
Quiñones et al. (2015)	Community (100)	26.9	NR	NR	PCL-C	Combat	SY + supportive therapy	MoBI	WL + supportive therapy; Passive	Significant difference in improvement in self-reported (PCL-C) PTSD symptoms between groups (*p* < 0.05). PTSD treatment effect of *d* = 1.15 within the SY group and between the effect of *d* = 0.73.
Reinhardt et al. (2018)	Veterans (51)	11.76	47.76	13.77	SCID; CAPS	NR	KY	MoBI	NT; Passive	KY group showed reductions in clinician-rated (CAPS) PTSD symptom levels to moderate PTSD/threshold range for the previous week but not for the previous month. No reductions in the NT control group.
Somohano and Bowen (2022)	Community (83)	100	34.67	9.27	PCL-5; primary clinician	NR	Trauma-Integrated Mindfulness-Based Relapse Prevention (38)	MiBI	Mindfulness-Based Relapse Prevention (45); Active	Significantly greater reductions in PTSD symptoms in control versus TI-MBRP at post-intervention (*p* = 0.016) and 1-month follow-up (*p* = 0.022). Significant reduction in PTSD symptoms at 12-month follow-up in both conditions (*p* < 0.001).
van der Kolk et al. (2014)	Community (64)	100	42.9	12.0	CAPS-IV; DTS	NR	Trauma-informed HY (32)	MoBI	Women’s HE (32); Active	Significant between-groups difference in pattern of change in clinician-rated (CAPS) PTSD symptoms (*d* = −0.34). Both groups exhibited significant reductions in PTSD. Per clinician-rated (CAPS) PTSD assessment, at post-treatment, 16 of 31 participants in the HY condition no longer met the criteria for PTSD compared to 6 of 29 in the HE condition. Large effect size for a decrease in PTSD symptoms in HY condition (*d* = 1.07) and medium-to-large for HE condition (*d* = −0.037). Symptoms at mid-course, but only the HY group maintained improvements through post-course.

Citation	Population (*N*)	% Female	Age *M* (*SD*)		Diagnostic method	Trauma type	MBI (*N*)	MBI type	Control (*N*)	Main findings
Wahbeh et al. (2016)	Veterans (114)	5.88	52.1	12.4	CAPS	NR	MM (27); MM + SB (25)	MMI	SB (25); SQ (25); Active	No significant between-group differences. Significant within-group improvement in self-reported (PCL) PTSD symptoms in MM condition (*p* = 0.05).
Yi et al. (2022)	Community (94)	100	41.5	14.5	DSM-5 criteria	Motor vehicle accidents	Kripalu yoga (47)	MoBI	Group discussion (47); passive	Significant reductions in self-report (IES-R) PTSD symptoms in yoga versus control (*p* = 0.01). Significantly lower intrusion (*p* = 0.004) and avoidance (*p* = 0.04) symptoms in yoga versus control at post-intervention.

RCT, randomized-controlled trial; MBI, mind–body intervention; CAPS-IV, Clinician-Administered PTSD Scale for DSM-IV; MRP, mantra repetition program; MMI, meditation- and mantra-based intervention; TAU, treatment as usual; PCL, posttraumatic checklist; NR, not reported; SCID, Structured Clinical Interview for DSM-5; MBSR, mindfulness-based stress reduction; MiBI, mindfulness-based intervention; SKY, Sudarshan Kriya Yoga; PCGT, present-centered group therapy; MoBI, movement-based intervention; WL, waitlist; PCL-M, posttraumatic checklist for military; WLP, wellness lifestyle program; HYP, holistic yoga program; PCL-17, posttraumatic checklist 17 items; KnY, kundalini yoga; PCL-C, posttraumatic checklist for civilians; PSS-I, posttraumatic symptom scale – interview; VC, Veteran.Calm psychoeducation; TM, transcendental meditation; HE, health education; PE, prolonged exposure; SY, satyananda yoga; KY, kripalu yoga; NT, no treatment; DTS, Davidson Trauma Scale; MM, mindfulness meditation; SB, slow breathing; SQ, sitting quietly.

Thirteen studies showed high ROB, suggesting that their findings might be influenced by factors such as attrition and inadequate treatment fidelity monitoring. Six studies were rated as having “some concerns,” and seven were rated as having low ROB.

### 3.1 Mindfulness-based interventions (MiBIs)

#### 3.1.1 Intervention and sample characteristics

Eight of the 26 studies evaluated MiBIs for PTSD. Six did so in veteran samples and two used community samples. Of these eight MiBI studies, six investigated Mindfulness-Based Stress Reduction (MBSR), a group-based intervention of 8–10 weekly sessions (Grossman et al., 2004). MBSR is a common MiBI and has been applied across populations and clinical presentations (Grossman et al., 2004). Of the six RCTs of MBSR, three of them (Polusny et al., 2015; Bremner et al., 2017; Davis et al., 2019) used person-centered group therapy (PCGT) as the control condition. Two studies of MBSR (Kearney et al., 2013, 2016) used treatment-as-usual (TAU). In both of these studies, the MBSR condition also included TAU. The remaining MBSR study used an active wellness control group (Gallegos et al., 2020). Another MiBI study (Niles et al., 2012) compared an MBSR-based telehealth intervention to a psychoeducation control. Of these studies, two were rated as having low ROB (Polusny et al., 2015; Bremner et al., 2017), two had some concerns (Kearney et al., 2016; Davis et al., 2019), and four had high ROB (Niles et al., 2012; Kearney et al., 2013; Gallegos et al., 2020; Somohano and Bowen, 2022) (see Table 5).

**Table 5:** Risk of bias domain ratings for individual studies.

Study	1. Randomization	2. Deviation from intervention	3. Missing outcome data	4. Measurement of outcome	5. Selection of reported result	Overall ROB rating
Bellehsen et al. (2022)	Low	Low	Low	Low	Low	Low
Bormann et al. (2018)	Low	Low	Low	Low	Low	Low
Bormann et al. (2013)	Low	Low	Low	Low	Low	Low
Bormann et al. (2008)	Some concerns	Low	Low	Low	Low	Some concerns
Bremner et al. (2017)	Low	Low	Low	Low	Low	Low
J.Carter et al. (2013)	Some concerns	Low	Low	Low	Low	Some concerns
Davis et al. (2019)	Low	Low	Some concerns	Low	Low	Some concerns
Davis et al. (2020)	Low	High	Some concerns	High	Low	High
Gallegos et al. (2020)	Low	Low	High	Low	Low	High
Gibert et al. (2022)	Some concerns	Low	Low	High	Low	High
Goldstein et al. (2018)	Some concerns	Low	Low	Low	Low	Some concerns
Jindani et al., 2015	Some concerns	Some concerns	Low	High	Low	High
Kearney et al. (2021)	Low	Low	Some concerns	Low	Low	Some concerns
Kearney et al. (2016)	Some concerns	Low	Low	Low	Low	Some concerns
Kearney et al. (2013)	Some concerns	Low	Low	High	Low	High
Kelly et al. (2021)	Low	Low	High	Low	Low	High
Lang et al. (2019)	Low	High	Some concerns	Low	Low	High
Nidich et al. (2018)	Low	Low	Low	Low	Low	Low
Niles et al. (2012)	Some concerns	Some concerns	Low	High	Low	High
Polusny et al. (2015)	Some concerns	Low	Low	Low	Low	Low
Quiñones et al. (2015)	Some concerns	Some concerns	Low	High	Low	High
Reinhardt et al. (2018)	Some concerns	High	High	Low	Low	High
Somohano and Bowen (2022)	Low	Low	High	High	Low	High
van der Kolk et al. (2014)	Some concerns	Low	Low	Low	Low	Low
Wahbeh et al. (2016)	Low	Some concerns	High	High	Low	High
Yi et al. (2022)	Some concerns	High	Low	High	Low	High

ROB, Risk of bias.

#### 3.1.2 Outcome assessment, control conditions, and findings for mindfulness-based stress reduction (MBSR) studies

Two studies of MBSR with PCGT control groups evaluated PTSD symptoms via the Clinician-Administered PTSD Scale (CAPS; Bremner et al., 2017; Davis et al., 2019). Bremner et al. (2017) found that only MBSR participants showed significant reductions in PTSD symptoms that were maintained at 6-month follow-up. However, Davis et al. (2019) found that PTSD symptoms were significantly reduced in both MBSR and PCGT conditions with no between-group differences. The MBSR condition showed significantly reduced self-reported PTSD symptoms, assessed via the Posttraumatic Checklist (PCL) compared to PCGT, but this difference was not maintained at the 2-month follow-up. The final study with a PCGT control (Polusny et al., 2015) found significant reductions in self-reported PTSD symptoms in both conditions from pre-intervention to 4-month follow-up, though the MBSR condition showed a significantly greater reduction.

Two MBSR studies with the TAU control conditions reported intent-to-treat (ITT) and completer analyses, though they described different findings. One of these studies (Kearney et al., 2013) found no effect of MBSR on PTSD symptoms post-treatment, and completer analyses showed no between-group differences at post-treatment or 4-month follow-up. This study reported clinically meaningful changes in PTSD symptoms. However, there was no significant difference between conditions in the percentage of participants with a clinically meaningful change in PTSD symptoms post-treatment. Kearney et al. (2016) used a semi-structured clinical interview assessment, the PTSD Symptom Scale-Interview (PSS-I). This study reported significant improvement in PTSD symptoms in the MBSR + TAU condition versus TAU alone at post-treatment and follow-up in a completer analysis. ITT analyses found similar results at post-treatment, though the difference was not maintained at follow-up. The final MBSR study (Gallegos et al., 2020) reported ITT analyses and found no significant between-groups differences for PTSD symptoms as indicated by the PCL-5. However, this study also reported significant decreases in PTSD symptoms severity within the MBSR group post-intervention but not in the control condition.

#### 3.1.3 Outcome assessment, control conditions, and findings for non-mindfulness based stress reduction (MBSR) studies

Niles et al. (2012) measured PTSD symptoms via the CAPS for DSM-IV and PCL-Military Version (PCL-M). Results indicated that there were significant pre- to post-intervention reductions in PTSD symptoms in the MBSR-based telehealth condition and no change in PTSD symptoms in the control condition. Results were similar for the CAPS and PCL-M measures at post-intervention and 6-week follow-up. The authors reported no time-by-group interaction for any results. Somohano and Bowen (2022) measured PTSD symptoms via PCL-5 in a study of Trauma-Integrated Mindfulness-Based Relapse Prevention (TI-MBRP). These authors reported significant improvement in PTSD symptoms across conditions. They also found a slightly larger improvement in the control condition (unmodified MBRP) compared to TI-MBRP at post-intervention and 1-month follow-up.

#### 3.1.4 Strength of evidence

The strength of evidence for MiBIs is rated as low, according to GRADE. This rating took into account passive and active comparators, conflicting findings, the small number of studies, small sample sizes in some studies, and moderate ROB rating for the majority of MiBIs studies.

### 3.2 Meditation- and mantra-based interventions (MMIs)

#### 3.2.1 Intervention and sample characteristics

Eight of the 26 primary studies examined MMIs, and all did so within veteran samples. Of these eight studies, two studied a mantra-based intervention compared to the TAU (Bormann et al., 2008, 2013), and one compared a mantra-based intervention to PCGT (Bormann et al., 2018). One study (Lang et al., 2019) evaluated compassion meditation compared to Veteran.Calm, a mind–body intervention emphasizing relaxation. Kearney et al. (2021) compared a loving-kindness meditation intervention to CPT. Nidich et al. (2018) investigated transcendental meditation (TM) compared to PE and PTSD health education control groups, and Bellehsen et al. (2022) compared TM to TAU. The final MMI study (Wahbeh et al., 2016) investigated two MMIs (body scan mindfulness meditation and mindful awareness of the breath) in two control groups (slow breathing with biofeedback and sitting quietly). Of these studies, four were rated as low ROB (Bormann et al., 2013, 2018; Nidich et al., 2018; Bellehsen et al., 2022), two were rated as some concerns for ROB (Bormann et al., 2008; Kearney et al., 2021), and one was rated as high ROB (Wahbeh et al., 2016).

#### 3.2.2 Outcome assessment, control conditions, and findings

Bormann et al. (2008) compared a mantra intervention to a delayed-treatment control. They found large between-groups effect sizes for reduced PTSD symptom severity. A follow-up study Bormann et al. (2013) compared a mantra repetition program (MRP) as a supplement to TAU versus a TAU alone condition. Their ITT analysis found that MRP + TAU resulted in significantly reduced patient- and clinician-reported PTSD symptoms at post-treatment compared to the TAU condition. Bormann et al. (2018) evaluated an MRP compared to PCGT and found that participants in the MRP condition showed significantly greater improvement in total CAPS scores at post-intervention and 2-month follow-up compared to the control. Lang et al. (2019) found greater improvement in PTSD symptoms in the compassion meditation group compared to Veteran calm. Kearney et al. (2021) found that CAPS-5 scores were lower in the loving-kindness condition at post-intervention and at the 3-month and 6-month follow-up. However, they also reported no significant between-groups differences in CAPS-5 scores. Nidich et al. (2018) found that TM was non-inferior to PE, and both transcendental meditation and PE were superior to the health education control. Bellehsen et al. (2022) reported that decreases in CAPS-5 and PCL-5 scores were significantly larger in the TM group compared to the TAU. Wahbeh et al. (2016) reported that participants in the meditation arms showed pre- to post-intervention improvement in PTSD symptoms, though there were no significant between-groups effects. Furthermore, this study found a significantly higher perception of improvement in PTSD symptoms in meditation conditions compared to controls.

#### 3.2.3 Strength of evidence

The strength of evidence for studies of MMIs was rated as low per GRADE primarily due to moderate ROB across studies, use of passive comparators, and a small overall number of studies. Furthermore, some studies of MMIs showed small effect sizes or contradictory findings.

### 3.3 Movement-based interventions (MoBIs)

#### 3.3.1 Intervention and sample characteristics

Ten of the 26 studies examined MoBIs. Five of them examined MoBIs in community samples, four in veteran samples, and one in a mixed community and veteran sample. Of these ten MoBI studies, eight investigated yoga interventions. Carter et al. (2013) evaluated Sudarshan Kriya Yoga (SKY), a standardized yoga program adapted for veterans, compared to a waitlist control. SKY consists of 22 hours of guided yoga and a 2 hour group yoga session weekly for the first month and then monthly for five months. Davis et al. (2020) investigated a 16-week holistic yoga program (HYP) designed to address hyperarousal in PTSD compared to a wellness lifestyle attention control, matched to HYP for physical activity. Jindani et al. (2015) compared a Kundalini Yoga (KY) intervention to a waitlist control. Quiñones et al. (2015) compared Satyananda Yoga (SY) to a waitlist control. Reinhardt et al. (2018) evaluated a yoga intervention based on Kripalu Yoga compared to a no-intervention control. Van der Kolk et al. (2014) studied a trauma-informed yoga (TIY) intervention compared to a supportive women’s education class. This control emphasized self-efficacy and did not include trauma-related content. Kelly et al. (2021) investigated a trauma-sensitive yoga intervention compared to CPT. Yi et al. (2022) evaluated the effect of a Kripalu yoga intervention for women diagnosed with PTSD resulting from motor vehicle accidents compared to a group discussion and board game control. Gibert et al. (2022) investigated the effects of a meditative scuba diving intervention (Bathysmed) compared to a multisport control, which involved participants engaging in outdoor activities such as hiking or canoeing. The remaining MoBI study (Goldstein et al., 2018) evaluated an integrative exercise program that combined traditional exercise with mindfulness-based practices tailored to veterans with PTSD. This intervention was compared to a waitlist control group. Of the eight MoBI studies, one was rated as low ROB (van der Kolk et al., 2014), three were rated as having some concerns for ROB (Carter et al., 2013; Goldstein et al., 2018; Davis et al., 2020), and six were rated as high ROB (Jindani et al., 2015; Quiñones et al., 2015; Reinhardt et al., 2018; Kelly et al., 2021).

#### 3.3.2 Outcome assessment, control conditions, and findings

Carter et al. (2013) reported significant reductions in PTSD symptoms in the SKY condition from pre- to post-treatment compared to control, which continued at a 6-week follow-up. Following the study, the waitlist group was allowed to participate in the same treatment, after which significant pre-, post-, and follow-up reductions in PTSD symptoms were reported. Davis et al. (2020) compared a HYP to a wellness lifestyle program (WLP). Both the HYP and WLP conditions showed significant improvements in PTSD symptoms from pre-intervention to 7-month follow-up. The HYP group demonstrated greater improvement post-treatment, though this difference was not observed at the 7-month follow-up. Self-report PTSD symptoms measured by the PCL-5 were significantly improved in the HYP at mid-treatment (eight weeks) and post-treatment compared to WLP. These differences were not observed at the 7-month follow-up. Jindani et al. (2015) compared a KY intervention to a waitlist control. Both groups demonstrated significant improvements in PTSD symptoms, and the KY condition exhibited significantly lower symptoms at post-treatment. Quiñones et al. (2015) compared a SY intervention to a waitlist control. This study reported significant decreases in pre- to post-intervention PTSD symptom scores in both groups. However, the SY condition demonstrated a large effect size for this reduction, whereas the waitlist control showed a small-to-medium effect. This study also examined the impact of the SY intervention on PTSD symptom clusters. In this analysis, the SY group showed large effect sizes for all clusters, while the control condition demonstrated small-to-medium effect sizes. Reinhardt et al. (2018) examined a yoga intervention based on Kripalu Yoga compared to a no-intervention control. The Kripalu yoga condition showed a reduction from severe-to-moderate PTSD symptom severity, while the control condition showed no change in severity. Both conditions demonstrated significant reductions in re-experiencing symptoms but no between-group differences. Van der Kolk et al. (2014) compared a TIY intervention to a supportive women’s health education group (HE). They found that 52% of participants in the TIY condition no longer met the criteria for PTSD post-intervention. Both groups showed a significant pre- to post-intervention reduction in PTSD symptoms; the TIY condition demonstrated a large effect size, and the HE group showed a medium-to-large effect size. Similarly, both conditions showed significant reductions in CAPS from pre- to mid-intervention time points, though the pattern of improvement differed between the groups: Only the TIY maintained this pattern from mid- to post-treatment. Kelly et al. (2021) evaluated a trauma-sensitive yoga intervention for CPT and found that CAPS-5 and PCL-5 scores decreased significantly over time. However, these authors reported no between-group differences, except for PTSD symptom Cluster B (i.e., intrusive symptoms) at 3-month follow-up. At this time point, CPT was found to result in significantly lower scores than the trauma-sensitive yoga intervention. Yi et al. (2022) studied a Kripalu yoga intervention compared to a group discussion and board game passive control. These authors found significant improvement in overall PTSD symptoms in the yoga condition versus control, as well as significant reductions in intrusion (i.e., re-experiencing symptoms) and avoidance symptoms in the yoga condition versus control. No significant between-group findings were noted at 3-month follow-up. Gibert et al. (2022) investigated a meditative scuba diving intervention (Bathysmed) against a multisport control intervention. They reported non-significant improvements in overall PTSD symptoms in the Bathysmed condition compared to the control condition. They also found that Bathysmed significantly improved intrusive symptoms compared to control 1-month post-intervention. No between-group differences were noted at 3-month follow-up. Goldstein et al. (2018) compared an integrative exercise program, combining traditional exercise with mindfulness-based practices tailored to veterans with PTSD, to a waitlist control. The integrative exercise condition showed a greater reduction in symptoms pre- to post-treatment than control. They also explored symptom clusters and found that the intervention condition demonstrated greater improvement in re-experiencing and avoidance/numbing than the waitlist control.

#### 3.3.3 Strength of evidence

Studies of MoBIs were rated as moderate strength of evidence per GRADE (Granholm et al., 2019). All studies favored MoBIs compared to controls, and the overall sample size for this category was relatively high (*N* = 881). However, six studies had high ROB, and only two used an active comparator, reducing the overall strength of evidence and confidence in their findings.

### 3.4 Physiological outcomes and findings

Despite a promising body of literature demonstrating the benefits of MBIs for physiological outcomes (Kwok et al., 2016; Buric et al., 2017; Stefanopoulou and Grunfeld, 2017), studies of MBIs for PTSD have lacked physiological or objective outcome measurement. Of the 26 primary studies included in this review, only three included such outcome measures.

Bremner et al. (2017) conducted positron emission tomography and found that MBSR and PCGT conditions showed differential brain activation in response to combat trauma-related images. Specifically, the authors found increased activation in regions related to physiological stress reactivity in the MBSR condition compared to PCGT.

Wahbeh et al. (2016) evaluated mindfulness meditation for PTSD symptoms via three objective mechanistic pathways: Autonomic nervous system activity (heart rate and heart rate variability), frontal cortex activity (attentional network tasks [ANT] conflict effect), and hypothalamic–pituitary–adrenal axis activity (cortisol awakening response). This study also measured resting respiration rates across conditions. The authors found that resting respiration decreased in the meditation groups compared to controls, but heart rate, heart rate variability, and ANT outcomes did not differ between conditions. They also reported that awakening cortisol was lower pre- to post-intervention in the mindfulness condition but did not differ between groups.

Gallegos et al. (2020) explored the effect of MBSR on physiological stress regulation, as indicated by the root mean square of successive differences (RMSSD) between heartbeats. RMSSD is a metric of heart rate variability and indicates vagally mediated autonomic control over the heart (Gallegos et al., 2020). Physiological data were collected over a 5-min period during which participants were seated quietly and then during a trauma imagery stressor task. The authors reported a non-significant increase for participants in the MBSR conditions for heart rate variability from baseline to 3-month follow-up post-intervention and a non-significant decrease in heart rate variability in the control condition over the same period.

## 4 Discussion

This systematic review investigated the effectiveness of MBIs for PTSD in adults. Twenty-six studies met the inclusion criteria. MBIs were divided into three categories: mindfulness-based interventions (MiBIs), meditation- and mantra-based interventions (MMIs), and movement-based interventions (MoBIs). This review examined the use of active or passive controls. Previous reviews of MBIs for PTSD identified a lack of comparator interventions as a primary limitation (Taylor et al., 2020). Although this review was limited to RCTs and findings suggest that MBIs may be useful for PTSD when compared to some passive or active control conditions, there continues to be a shortage of RCTs comparing MBIs to “first-line” PTSD interventions. An exception in this review is Nidich et al. (2018), which compared a transcendental meditation intervention to PE and found that both significantly reduced PTSD symptoms and were superior to a health education control condition. Another exception is Kelly et al. (2021), which evaluated a Kripalu yoga intervention compared to cognitive processing therapy. These authors found significant within-group reductions in PTSD symptoms, and only between-group differences in Cluster B (intrustion) symptoms at 3-month follow-up. Identifying alternative approaches can be important for patients who are reluctant to engage in exposure-based interventions or are left with residual symptoms (Bradley et al., 2005; Kim et al., 2013; Steenkamp et al., 2015). Replication of this study and additional comparisons of MBIs to “first-line” interventions are necessary to clarify their relative utility.

Increased objective outcome measurement is also important to advancing this research. The three studies in this review that included objective outcomes reported promising preliminary findings. Bremner et al. (2017) found increased activation of regions associated with physiological stress reactivity in an MBSR condition compared to PCGT. Wahbeh et al. (2016) found a decrease in resting respiration in the meditation conditions and decreased pre- to post-mindfulness meditation waking cortisol. Gallegos et al. (2020) found that MBSR non-significantly improved participants’ heart rate variability, whereas the controls showed worsened heart rate variability.

We found moderate-strength evidence for MoBIs in treating PTSD symptoms when evaluated alongside a passive comparator. All seven RCTs in this category favored MoBIs over the control. However, five of the seven showed high ROB, limiting confidence in their findings. We found low strength of evidence for MoBIs versus active comparators and for MMIs versus both active and passive comparators, largely due to moderate ROB and a small number of studies in each category. The strength of evidence for MiBIs versus both active and passive comparators was very low, primarily due to conflicting results.

“First-line” treatments have demonstrated efficacy across studies (Resick and Schnicke, 1992; Ahrens and Rexford, 2002; Foa and Rauch, 2004; Bradley et al., 2005; Monson et al., 2006; Even-Chen and Itzhaky, 2007; Rauch et al., 2009; Berninger et al., 2010; Steenkamp et al., 2015; Kehle-Forbes et al., 2016). As such, the field is cautious about adopting new interventions. However, some patients may hesitate to engage in cognitive- or exposure-based interventions, drop out, or have difficulty adhering to treatment (D'Andrea and Pole, 2012; Niles et al., 2018). For these individuals, having options is crucial. More trials investigating MBIs in direct comparison to “first-line” interventions are required to evaluate their comparative efficacy. Furthermore, greater use of objective outcome measures will help illuminate biological pathways and assist in honing MBIs for PTSD.

### 4.1 Limitations

There are some notable limitations to this review. Studies were limited to those identified in the PTSD-Repository (O'Neil M. E. et al., 2020; O'Neil M. et al., 2020; O'Neil et al., 2022) and although this database is comprehensive for RCTs of PTSD interventions in adults, some potentially relevant research may have been excluded due to the inclusion criteria for that database (e.g., excluding studies of adults with subthreshold PTSD). Second, there is a high heterogeneity of assessment tools used for PTSD diagnosis and symptom change. While many studies utilized the CAPS, a gold standard assessment tool (Hunt et al., 2018), some used other semi-structured interview assessments [e.g., SCID (Goldstein et al., 2018); MINI 7.0.0 (Kelly et al., 2021)] and others used self-report measures [e.g., PCL; (Jindani et al., 2015); PSS-I (Kearney et al., 2016)]. Past research has cautioned against the sole use of self-rated measures of PTSD (McDonald and Calhoun, 2010) and has distinguished between the constructs assessed by each of these measures. Among the studies in this review, findings did not differ based on the outcome assessment tool. Risk of bias assessment and, therefore, the overall strength of evidence did account for potentially increased bias in self-report measures. Our confidence in findings could be strengthened by future studies utilizing clinician-administered PTSD assessments as well as RCTs that evaluate the effectiveness of mindfulness through resonance measures of neural activity (King et al., 2016).

Previous studies have also argued that the relationship between the type or amount of trauma sustained and the presentation of PTSD is not well-enough accounted for in the literature (Smith et al., 2016). Trauma type also appears to impact the efficacy of PTSD interventions. Similarly, although the DSM-5 (American Psychiatric Association, 2013) allows PTSD diagnoses based on cumulative trauma, studies of PTSD often identify an “index” trauma that may misrepresent the experience of individuals exposed to chronic trauma (Smith et al., 2016). This may be particularly relevant in light of the psychological impacts of the COVID-19 pandemic (Cavalera et al., 2023), especially among communities of color (Cooper and Williams, 2020; Vázquez et al., 2023). Among the studies in this review, findings did not differ significantly based on the type(s) of trauma experienced by participants. Overall, heterogeneity across studies in areas such as sample characteristics and outcome assessment should be considered in future reviews because of the potential impact of these factors on PTSD outcomes.

### 4.2 Conclusion

The studies included in this systematic review suggest that MBIs are likely effective for improving PTSD outcomes in some individuals; however, there are not many head-to-head trials comparing MBIs to “first-line” interventions. The findings are mixed for many categories of MBIs, and the strength of this body of evidence is generally low to moderate. This systematic review of the literature found that MoBIs had relatively higher (moderate) strength of evidence compared to MMIs and MiBIs and showed the most consistently favorable outcomes. High-quality, larger trials comparing MBIs to “first-line” evidence-based treatments for PTSD are needed to increase the strength of evidence and further clarify which, if any, of these interventions warrant broader dissemination. More high-quality trials utilizing active comparators are also necessary to increase confidence in findings, primarily due to the potential for biased findings when participants are aware of their passive comparator group status. Furthermore, future studies should include an objective assessment of PTSD and associated physiological outcomes to overcome inherent limitations of self-report and other subjective rating systems. Additionally, when more head-to-head comparisons of MBIs and “gold standard” or “first-line” treatments are available, meta-analyses should be conducted to more directly investigate the comparative efficacy of these interventions. Finally, given the proliferation of recent systematic reviews and meta-analyses in this area, future evidence synthesis work evaluating mind–body interventions for PTSD should consider whether enough novel research has been conducted to significantly add to our knowledge. Similarly, a review of reviews and meta-analyses may be warranted and more appropriate to answer questions of interest.

## Author contributions

JK assisted in conducting the initial search and data abstraction, and authored the manuscript. VS assisted in authorship of the manuscript and interpretation of findings. BZ assisted in authorship of the manuscript and interpretation of findings. MO’N conducted the initial search, portions of the data abstraction, and interpretation of findings, and assisted in authorship and construction of the manuscript.
